# Nar1 binds the cytosolic iron–sulfur cluster assembly targeting complex *via* bipartite interactions

**DOI:** 10.1016/j.jbc.2026.113297

**Published:** 2026-06-25

**Authors:** Jackson V. Ho, Anastasiya Buzuk, Melissa D. Marquez, Beatrice Wang, Deborah L. Perlstein

**Affiliations:** Department of Chemistry, Boston University, Boston, Massachusetts, USA

**Keywords:** iron–sulfur protein, iron metabolism, molecular modeling, protein-protein interaction, protein complex, protein structure, protein targeting

## Abstract

The cytosolic iron-sulfur cluster assembly (CIA) pathway maturates essential nuclear and cytosolic Fe–S proteins required for genome maintenance and cellular metabolism. Nar1 (also called CIAO3 or IOP1) is a conserved Fe–S protein that connects the early and late steps of the CIA pathway, yet the molecular basis for its proposed function as a metallocluster carrier remains poorly defined. In particular, the interactions responsible for Nar1 recruitment to the CIA targeting complex (CTC) during cluster delivery remain unknown. Here, we define the molecular basis for Nar1 recruitment to the CTC using biochemical reconstitution, quantitative protein-protein interaction assays, and AlphaFold modeling. Our data reveal that Nar1 binds the CTC through two distinct interfaces. A primary interface comprises an electrostatic interaction that anchors Nar1 to a conserved acidic surface on the Cia1 subunit of the CTC and a secondary interface involves binding of Nar1’s divergent targeting complex recognition peptide at the Cia1-Cia2 interface. Thus, Nar1 engages a conserved CTC surface that serves as a recruitment platform for multiple binding partners, including CIA clients. Computational structural models position the putative Fe–S cluster donor site of Nar1 adjacent to a proposed acceptor site on Cia2, suggesting that this bipartite binding mechanism positions Nar1 for potential transfer of an Fe–S cluster to the targeting complex. Together, these findings resolve conflicting models for Nar1 recruitment and establish a mechanistic framework for understanding how the CTC engages multiple binding partners during cytosolic iron-sulfur protein maturation.

Iron-Sulfur (Fe–S) proteins participate in a wide variety of fundamental cellular processes across all domains of life (1, 2, 3, 4). Due to the fragility of these cofactors in aerobic environments and the toxicity and scarcity of free iron and sulfide *in vivo*, the maturation of Fe–S enzymes requires a complex network of conserved and essential metallocofactor biogenesis factors that assemble, traffic, and target nascent Fe–S cofactors, facilitating maturation of apo-client proteins. In eukaryotes, Fe–S cluster biogenesis occurs across multiple subcellular compartments, operating in the mitochondria, in cytosol and nucleus, and, when presents, plastids (1, 2, 3). The cytosolic iron-sulfur cluster assembly (CIA) system maturates cytosolic and nuclear Fe–S proteins, including primase, DNA and RNA polymerases, helicases and glycosylases. Because these nuclear Fe–S proteins are critical for DNA metabolism and maintenance, cells rely on highly controlled CIA pathway to assemble and deliver their metalloclusters. Consequently, inhibition of CIA leads to genome instability, defects in telomere maintenance, increased mutation rates and replicative stress, and contributes to diseases including neuromuscular degeneration and cancer (5, 6, 7, 8). Molecular insights into diseases associated with disruption of the CIA pathway require an improved understanding of the fundamental biochemistry underpinning CIA function, which remains generally understudied relative to other Fe–S cluster biogenesis machineries.

In the late stages of the CIA pathway, Fe–S clusters are delivered to clients *via* the CIA targeting complex (CTC; Fig. 1*A*) (1, 9, 10, 11, 12). This multiprotein complex is proposed to act as the central delivery hub, accepting a nascent cluster from the upstream trafficking factor Nar1 (also known as CIAO3, IOP1 or NARFL in humans, Table S1) and subsequently delivering the metallocluster to a CIA client (13, 14). Structural and biochemical studies have begun to reveal how the CTC subunits Cia1 (CIAO1), Cia2 (CIAO2B/FAM96B/MIP18 in humans, herein called *Hs*Cia2b, Table S1), and Met18 (MMS19 in humans) assemble to form the CTC and recruit CIA clients (10, 11, 12, 15, 16). Insights into the structure of the CTC have advanced recently, with reported X-ray crystal structures of Cia1, Cia2 and Met18 orthologs from yeast, fly and humans both alone and in complex (16, 17, 18, 19, 20). In addition, single particle cryo-EM reconstructions of the human CTC in complex with client proteins (∼9–12 Å resolution) have identified potential client-contacting regions, although not at atomic resolution (20). These structures and subsequent biochemical studies have led to the current working model for client recruitment to the CTC. Namely, this process requires at least two primary points of contact between the CTC and clients, one between a conserved surface along the side of Cia1’s third β-propeller domain and a second located in the N-terminal domain of Met18 (16, 19, 20).

**Figure 1:**
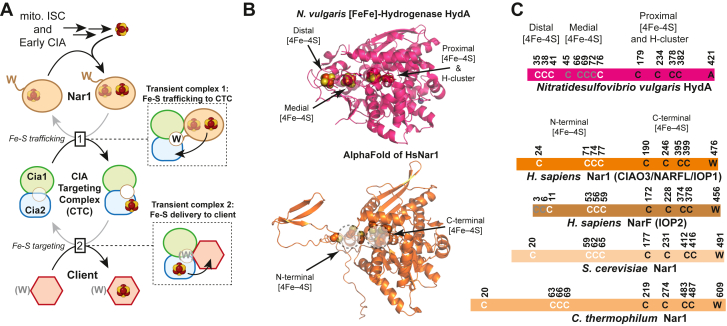
**Overview of the CIA pathway and the structural features of Nar1.***A,* schematic of the proposed Fe-S cluster transfer in the CIA pathway. The mitochondrial ISC system and early CIA factors synthesize a [4Fe-4S] cluster, which is transferred to Nar1. Next, Nar1 delivers the cluster to the CTC through formation of a transient complex (1), and interaction facilitated by Nar1’s C-terminal tail ending in a tryptophan (W) residue. Finally, the [Fe-S]-bound CTC transiently engages apo-clients (2), some of which end in a TCR peptide, to complete the maturation pathway. The MMS19/Met18 subunit of the targeting complex is omitted for clarity. *B,* structural comparison of [FeFe]-hydrogenase (PDB:1HFE) is shown as representative example) (51, 52, 53), and *Hs*Nar1, AlphaFold prediction (37). Proposed [Fe-S]-ligating cysteine residues of Nar1 are *spheres*. *Dotted circles* indicate approximate positions of Nar1’s N-terminal and C-terminal Fe-S binding sites. *C,* conserved cysteines of hydrogenase are shown, colored *white*, *gray* or *black* for the distal, medial and proximal [4Fe-4S] clusters. Nar1 homologs from *Hs, Sc* and *Ct* are shown, with their relative positioning and conservation with hydrogenases indicated by the position. The cysteines proposed to ligate the N-terminal and C-terminal Fe-S cluster of Nar1 are colored *white and black*, respectively. NarF sequences, found in certain vertebrates, including humans, have two additional conserved cysteine residues at their N-termini, colored *gray*. *Ct*, *Chaetomium.thermophilum;* Fe–S, Iron-Sulfur*; Hs*, *homo. sapiens*; TCR, targeting complex recognition.

There has also been recent progress on understanding how clients are identified by and recruited to the CTC. At least 20% of CIA clients contain a conserved targeting complex recognition (TCR) motif, a conserved C-terminal tripeptide ([LIM]-[DES]-[FW]) that is sufficient to recruit the targeting complex both *in vitro* and *in vivo* (Fig. 1*A*) (21). Biochemical studies revealed that the Cia1-Cia2 interface close to Cia1’s β-propeller domain is required for TCR peptide-based client recruitment (22). This proposal was corroborated by recent biochemical and crystallographic studies showing that this same interfacial site also accommodates an alternative targeting internal pentapeptide (23, 24). Despite these recent advances uncovering the mechanism of client recognition by the CTC, much less is known about the interactions that govern the CTC’s recruitment of Nar1, the putative CIA trafficking factor (Fig. 1*A*) (13, 25, 26, 27, 28).

Although Nar1 is widely proposed to function as a Fe–S cluster carrier in the CIA pathway through transiently binding and transferring a nascent cluster destined for cytosolic and nuclear Fe–S proteins (Fig. 1*A*), key aspects of its carrier function remain unresolved due to incomplete biochemical data and, at times, contradictory models. Early biochemical studies suggested that Nar1 interacts primarily with the Cia1 subunit of the targeting complex, whereas later studies indicated that additional components, such as Cia2 or the nascent Fe–S cluster itself, are required for stable CTC-Nar1 complex formation (27, 28, 29). The identification of a TCR-like motif at the C-terminus of Nar1suggested that Nar1’s tail could recruit the CTC (21, 22). The observation that Nar1 competes with TCR-bearing clients for CTC binding further supports the hypothesis that Nar1 is an Fe–S cluster carrier that connects the early- and late-acting steps of the CIA pathway (22). However, this model is challenged by proteomics studies indicating Nar1 can directly donate its Fe–S cluster to clients whereas other studies have concluded that ISCU/HSC20 complex, not Nar1, provides the Fe–S cluster to the CTC (28, 30, 31). These many contradictory findings collectively highlight the incomplete and fragmented nature of the literature regarding Nar1’s putative carrier function in the CIA pathway. Thus, despite Nar1’s central position in the CIA pathway, it remains one of the least understood proteins required for cytosolic and nuclear Fe-S protein maturation.

Nar1 itself is particularly intriguing because of its evolutionary relationship to [FeFe]-hydrogenases, ancient enzymes involved in hydrogen metabolism, which contain multiple Fe–S clusters that relay electrons to and from the catalytic H-cluster (Fig. 1, *B* and *C*) (32, 33). Nar1 retains eight conserved cysteine residues positioned analogously to residues ligating the proximal and medial [4Fe–4S] clusters of hydrogenase (25, 34). These Nar1 residues are therefore proposed to coordinate two [4Fe–4S] clusters, and possibly a third [2Fe–2S] cluster bound in place of the H-cluster (25, 34). Unlike the stably-bound Fe–S clusters of hydrogenase that function in electron transfer, at least one or more of the Nar1 metalloclusters are proposed to be transiently associated, allowing Nar1 to serve as an intermediate carrier proposed to link the early-acting CIA scaffold with the late-acting CTC. Structural insight into Nar1 has been limited, in part because isolation of fully cluster-loaded, homogeneous protein has proven difficult, leaving the molecular architecture of Nar1 and its interactions with the targeting complex poorly defined. The lability and oxygen sensitivity of these clusters, efforts to determine the number, nuclearity, and functional roles of these Fe–S cluster cofactors have been challenging to decipher. Additionally, some animals encode a second Nar1-like paralog, called NARF or IOP2 in humans, the role of which remains largely unknown (Fig. 1*C*) (14, 32). The challenges have led to persistent uncertainty regarding Nar1’s molecular function in the CIA pathway.

Another major barrier to understanding Nar1 function is the absence of structural information about Nar1 and how it interacts with the CTC. Although biochemical studies could presumably fill this gap, the lack of quantitative assays capable of defining how specific residues contribute to Nar1’s protein-protein interactions have further slowed progress toward defining the molecular mechanism by which Nar1 carries out its putative carrier function. Here, we address this gap by combining affinity copurification experiments, quantitative fluorescence anisotropy binding assays and AlphaFold3 modeling to define how Nar1 recruits the targeting complex. We show that Nar1 binds the CTC through a bipartite interaction mechanism involving a primary interaction site dominated by electrostatic interactions between a basic surface of Nar1 and an acidic patch on Cia1 and a secondary interaction site in which Nar1’s C-terminal tail engages the TCR peptide binding site at the Cia1-Cia2 interface. These findings resolve many of the seemingly contradictory findings related to Nar1’s protein-protein interactions and provide a mechanistic framework for understanding how Nar1 docks onto the targeting complex to enable Fe–S cluster delivery within the CIA pathway.

## Results

### Nar1 directly binds Cia1, and Cia2 further stabilizes formation of a defined three-protein complex

Previous studies have reached conflicting conclusions regarding whether Nar1 is recruited to the CIA targeting complex (CTC) through its interaction with Cia1 alone, or whether Cia2 also plays a critical role in mediating the interaction (22, 27). To resolve this discrepancy and arrive at a unified model through direct comparison of interaction behavior across evolutionarily distant systems, we examined interactions between Nar1, Cia1, and Cia2 using orthologs from fungi (*Chaetomium thermophilum, (Ct)*; *S. cerevisiae, Sc*) and humans (*Homo sapiens, (Hs*). Throughout this manuscript, species-specific proteins are indicated with two letter prefixes (*Ct, Sc, Hs*), whereas generic protein names using the budding yeast nomenclature are used when referring to the protein family.

Whereas fungi encode a single Cia2 homolog, metazoans possess two, Cia2a and Cia2b (10). Here, we focused on human Cia2a based on its reported interaction with Nar1 in proteomic studies, its ability to form a ternary Cia1-Cia2a-Nar1 complex *in vitro* and its robust heterologous expression and purification, enabling direct biochemical comparisons across evolutionarily distant orthologs (10, 18, 27).

The Nar1 proteins purified with partially assembled Fe–S cofactors (∼3–4 Fe atoms per polypeptide corresponding to ≤ 1x[4Fe–4S] cluster; see *Materials* and *Methods*), consistent with previous reports indicating incomplete cluster occupancy (25, 27, 34). Attempts to generate fully apo-protein resulted in destabilization, whereas attempts to increase cluster loading *via* chemical reconstitution or heterologous overexpression strains or growth media typically leading to increased Fe–S cluster incorporation were unsuccessful, as judged by unchanged spectroscopic signatures. Accordingly, the interaction studies were performed using as-isolated Nar1, which retained robust CTC binding activity.

**Figure 2:**
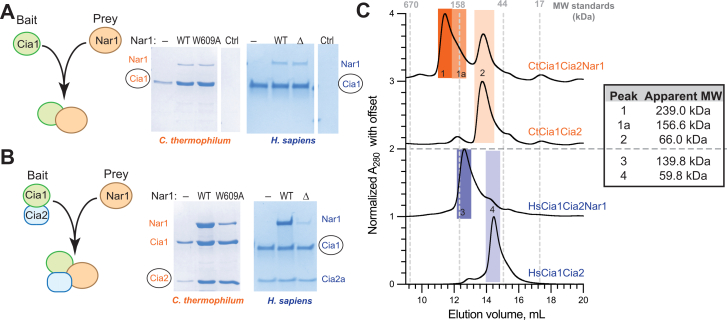
**Interaction of Nar1 with Cia1-Cia2.***A and B,* SDS-PAGE analysis (Coomassie staining) of affinity copurification assays examining the interaction of Nar1 with Cia1 alone *(A)* or the Cia1-Cia2 complex *(B)* using *Ct* (*orange*) and *Hs* (*blue*) orthologs. In each *panel*, the strep-tagged bait is *circled* and *negative controls* omitting the bait are labeled ctrl. The Nar1 variant present in each lane is indicated above the gel as follows: no Nar1 (−), WT Nar1 (wt), *Ct*Nar1-W609on of last five residues). Uncropped input and elution gels, including controls and molecular weight standards, are shown in Fig. S1. *C,* size exclusion chromatography analysis of the indicated Cia1-Cia2 and Cia1-Cia2-Nar1 complexes. Elution volumes of molecular weight standards are indicated with *dashed gray* lines. Major peaks are indicated by numbered *shaded boxes* (*Ct, orange, top; Hs, purple, bottom*). The apparent molecular weight of major species is indicated to the *right*. *Ct*, *Chaetomium.thermophilum; Hs*, *homo. sapiens*.

Affinity copurification assays using the thermophilic fungal (*Ct*) and human (*Hs*) Nar1 orthologs revealed that it associates with Cia1 in the absence of Cia2 (Figs. 2*A* and S1, *A*, *B*), corroborating our previous observations with the *Sc* proteins (22). Notably, inclusion of Cia2 reproducibly increased Nar1 recovery across all orthologs (Figs. 2*B* and S1, *A*, *B*). In contrast, *Sc*Cia2 alone showed little to no interaction with *Sc*Nar1 and inclusion of *Sc*Met18 did not promote *Sc*Nar1 association with *Sc*Cia2 (Fig. S1*E*). Thus, these data indicated that Cia1 is sufficient to bind Nar1, but Cia2 stabilizes the complex.

To probe the formation and stability of Cia1-Cia2-Nar1 (CCN) assemblies, we analyzed affinity copurified complexes by size exclusion chromatography (Fig. S2). *Ct*CCN eluted as a prominent high-molecular weight species (∼239 kDa) that was absent from the *Ct*Cia1-Cia2 control (Fig. 2*C*; Table S2). SDS-PAGE analysis confirmed the peak contained all three proteins (Fig. S2*B*), indicating formation of a stable higher-order complex upon addition of *Ct*Nar1. Although the elution profile suggests the presence of assemblies larger than a simple heterotrimer, the shoulder (∼157 kDa) approximates the predicted molecular weight of a 1:1:1 Cia1:Cia2:Nar1 complex (155.1 kDa). Similarly, the human CCN complex eluted as a discrete high-molecular weight species (∼140 kDa) containing all three proteins (Figs. 2*C* and S2*D*; Table S2), consistent with formation of at least a heterotrimeric species (predicted molecular weight 122.1 kDa). These results indicate both the *C. thermophilum* (*Ct*) and *H. sapiens* proteins can form stable CCN assemblies consistent with formation of heterotrimers as well as higher-order complexes, aligning with previous reports of the ability of the CTC or its individual subunits to form higher-order assemblies (19, 20, 35). Together, these data demonstrate that Nar1 directly associates with Cia1 and Cia2 and further stabilizes this three-protein complex, suggesting that Nar1 recruitment to the CTC involves contributions from both Cia1 and Cia2.

### Nar1’s divergent TCR motif recruits the CTC *via* a conserved pocket at the Cia1-Cia2 interface

Because our copurification studies indicated Nar1 recruits the CTC through a bipartite interaction involving both Cia1 and Cia2, we next sought to define the molecular features of this interface. Nar1 terminates in a conserved tryptophan that is essential for its role in the CIA (21), reminiscent of the targeting complex recognition (TCR) motif found at the C-terminus of many CIA clients (Fig. 1*A*). TCR peptides bind in a pocket formed at the Cia1-Cia2 interface and derive a substantial fraction of their binding energy from the terminal aromatic residue of this tripeptide motif (21, 24).

Despite this commonality, alignment of Nar1 orthologs diverged from the [LIM]-[DES]-[FW] targeting peptide consensus. In contrast to CIA clients featuring an acidic residue in the penultimate position of the TCR motif, none of the 16 Nar1 sequences derived from diverse eukaryotic organisms matched this consensus (Fig. S3*A*). To gain a comprehensive view of sequence conservation, we analyzed the C-terminal tripeptide of 631 Nar1 proteins. To our surprise, 45% of the sequences contained a basic lysine residue while only 4% had an acidic aspartate residue, which is the most frequently observed penultimate residue in canonical TCR peptides (Fig. S3*B*). Likewise, the most common antepenultimate residue in Nar1 is a valine (23%), whereas in canonical TCR motifs, the most frequently observed amino acids are Ile, Leu, and Met (21). This divergence suggested either Nar1 does not engage the TCR peptide pocket or does so through a modified binding mode distinct from that used by canonical TCR peptides in CIA clients.

To test whether Nar1’s C-terminus contributes to CTC recruitment, we examined how perturbation of Nar1’s C-terminus impacts its association with Cia1-Cia2. Deletion of the final five residues of *Hs*Nar1 (ΔTCR) or substitution of the terminal tryptophan in *Ct*Nar1 (W609A) markedly reduced copurification with their respective Cia1-Cia2 complexes to levels comparable to Nar1 recovered with Cia1 alone (Figs. 2, *A*, *B* and S1, *A*, *B*). In contrast, these variants had no detectable effect on the Nar1-Cia1 interaction in the absence of Cia2, indicating that Nar1’s C-terminal tail contributes to stabilization of the CCN complex through interactions involving Cia2.

**Figure 3:**
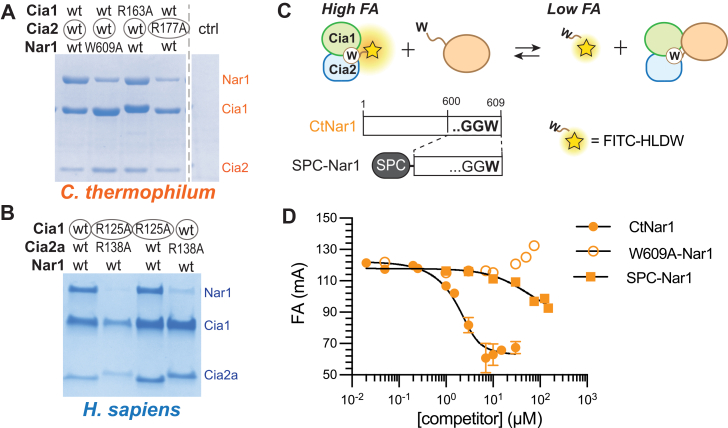
**Interaction of Nar1 with the TCR peptide binding site of Cia1-Cia2.***A and B,* SDS-PAGE analysis (Coomassie staining) of affinity copurification assays monitoring the interaction of Nar1 with variants of Cia1 and Cia2. A negative control, where bait was omitted is labeled Ctrl. Extended data, including inputs and controls, are provided in Fig. S1, *C* and *D*. *C and D,* scheme for the competitive FA assay *(C)* and the resulting data *(D)*. Competitive FA assay in which the FITC-HLDW probe (0.05 μM) is displaced from *Ct*Cia1-Cia2 by unlabeled competitor, including WT *Ct*Nar1, *Ct*Nar1-W609A, or SUMO peptide carrier for the *Ct*Nar1 C-terminus. The observed K_D_ values for *Ct*Nar1 and SUMO peptide carrier for the *Ct*Nar1 C-terminus were 0.4 μM and 190 μM, respectively. The W609A Nar1 variant did not displace the probe. FA, fluorescence anisotropy; TCR, targeting complex recognition.

To directly probe this interaction site, we substituted two conserved arginine residues within the TCR peptide binding pocket, one from Cia1 and one from Cia2, and assessed their impact on the formation of the CCN assembly (22, 23). Substitution of the Cia2-derived residue (R177A-*Ct*Cia2; R138A-*Hs*Cia2a) impaired Nar1 recruitment, whereas mutation of the Cia1-derived arginine (R163A-*Ct*Cia1; R125A-*Hs*Cia1) had little to no impact (Figs. 3, *A*, *B* and S1, *C*, *D*). This behavior contrasts sharply with CIA clients bearing canonical TCR motifs, which strongly depend on both arginine residues (22). These data indicate that Nar1’s C-terminus engages the TCR peptide binding pocket, likely through a non-canonical binding mode that depends strongly on Cia2, consistent with the divergence of Nar1’s tail from the TCR consensus.

To quantify the energetic contribution of Nar1’s C-terminus, we performed fluorescence anisotropy competition assay using a FITC-labeled tetrapeptide probe (FITC-HLDW; Fig. 3*C*) (22). Full-length *Ct*Nar1 displaced the probe from *Ct*Cia1-Cia2 with an apparent dissociation constant (*K*_*D*_) of 0.4 μM (Table S3), whereas the W609A variant failed to compete (Fig. 3*D*). Similar qualitative results were observed with the human proteins (Fig. S4), although protein aggregation precluded determination of a *K*_*D*_ value.

To isolate the energetic contribution from the Nar1 tail alone, we fused the final ten residues of *Ct*Nar1 to SUMO, generating a SUMO peptide carrier for the *Ct*Nar1 C-terminus (SPC-Nar1) (21, 22). SPC-Nar1 displaced the peptide probe with an IC_50_ over two orders of magnitude higher than the full-length *Ct*Nar1 (Fig. 3*D*, Table S3). In total, the qualitative and quantitative binding data demonstrate that Nar1’s C-terminal tail is sufficient to engage the TCR peptide pocket but contributes only modestly to the overall binding affinity, consistent with the divergence of the Nar1 tail from the canonical TCR consensus. Thus, Nar1 must exploit an additional binding surface beyond the Cia2-dependent interaction mediated by its C-terminal tail.

### A conserved electrostatic interface anchors Nar1 to the Cia1 subunit of the CTC

**Figure 4:**
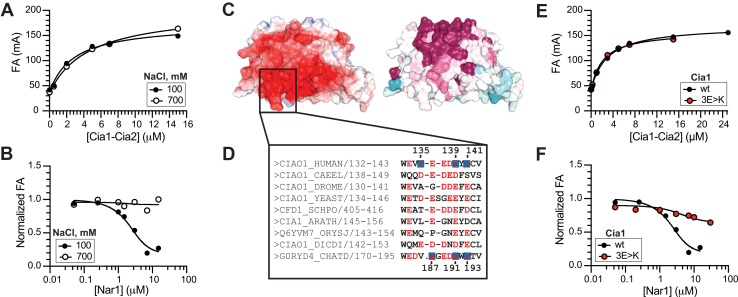
**A conserved acidic surface on Cia1 contributes to Nar1 binding.***A,* FA assay showing FITC-HLDW probe (0.05 μM) binding to *Ct*Cia1-Cia2 (0–15 μM) under low (100 mM) and high (700 mM) concentrations of NaCl and establishing that the ionic strength does not impact TCR peptide binding affinity. *B,* Competitive FA assay demonstrating the *Ct*Nar1 can displace the FITC-HLDW from *Ct*Cia1-Cia2 under low ionic strength (100 mM NaCl) conditions but fails to do so when the ionic strength of the buffer is raised (700 mM NaCl). *C,* surface representations of the *Hs*Cia1 crystal structure (PDB:3FM0) (17) colored by electrostatic potential (*left*) (54, 55), or residue conservation (*right; pink, conserved; cyan, variable*) using the alignment in Fig. S5 and colored using ConSurf server (50). *D,* sequence alignment of Cia1 orthologs highlighting region of the Asp/Glu-to-Lys substitutions in the 3E > K variants (*blue boxes*, numbering in human (*top*) and *C. thermophilum* (*bottom*) are indicated). *E,* FA assay (as in *Panel A*) showing that the *Ct*Cia1^3K>E^ variant (*red*) retains TCR peptide binding with comparable affinity as WT *Ct*Cia1. *F,* competitive FA assay (as in *Panel B*) demonstrates that the charge-swapped variant (*Ct*Cia1^3K>E^, *red*) is strongly disrupted in *Ct*Nar1 binding to the *Ct*Cia1-Cia2 complex. FA, fluorescence anisotropy; TCR, targeting complex recognition.

Having established that Nar1’s C-terminal tail engages the TCR peptide binding pocket but contributes modestly to the overall binding affinity of the CCN complex, we next sought to identify the dominant interaction surface mediating the Cia2-independent interaction. Guided by AlphaFold models (*vide infra*), we first tested whether electrostatic or hydrophobic forces drive Nar1-Cia1 complex formation by examining Nar1 binding to the CTC under conditions of increased ionic strength. Increasing the NaCl concentration from 100 to 700 mM abolished detectable *Ct*Nar1-mediated displacement of the FITC-HLDW probe from *Ct*Cia1-Cia2 without affecting the binding of the FITC-labeled TCR peptide (Fig. 4, *A* and *B*). These data suggest that Nar1's association with the CTC is strongly influenced by ionic interactions that are shielded as the buffer ionic strength is elevated.

To pinpoint the Cia1-Nar1 interaction interface, we focused on Cia1 because high-resolution crystal structures of multiple orthologs (fly, yeast, and human) are available, enabling confident identification of solvent-exposed surfaces for mutagenesis (16, 17, 20). Additionally, Cia1 has a WD40 β-propeller fold, a structurally conserved scaffold fold that is generally tolerant to substitutions at surface-exposed residues (17). Guided by the salt sensitivity of the Nar1-Cia1 interaction and its conservation across eukaryotes, we focused on blade 3 of the Cia1 β-propeller because this region was noted to be both highly conserved and negatively charged, and subsequent cryoEM reconstructions (8–12 Å) indicated this patch was positioned adjacent to CTC-bound client (Figs. 4*C* and S5) (16, 20). Because Nar1 and TCR-bearing clients compete for recruitment to the CTC (22), we hypothesized that this acidic patch could serve as the primary Cia1-centered docking site for Nar1.

To test this, we replaced three conserved glutamate residues on *Ct*Cia1 (E187, E191, E193) with lysine, generating the charge-reversed variant *Ct*Cia1^3E>K^ (Fig. 4*D*). These substitutions weakened *Ct*Nar1 binding to the *Ct*Cia1-Cia2 complex by ≥ 40-fold but did not affect Cia2 association nor TCR peptide binding affinity (Figs. 4, *E*, *F* and S6*A*; Table S3). The differential impact of these Cia1 variants on TCR peptide and Nar1 binding indicate that they selectively impact Nar1 recruitment as variants disrupting the Cia1-Cia2 complex are known to indirectly impact TCR peptide binding affinity (22). Analogous substitutions in *Hs*Cia1 similarly weakened binding to *Hs*Nar1 in affinity copurification assays (Figs. 4*D* and S6). Although the WT *Hs*Cia1 and its charge-swapped variant had similar melting temperatures, indicating that the substitutions were not strongly destabilizing, the limited stability and solubility of the human proteins relative to those from *C. thermophilum* prevented determination of dissociation constant. These quantitative and qualitative binding data indicate the conserved, acidic patch on the side of blade 3 of Cia1 as a major contributor to Nar1 recruitment to the CTC, defining the second, Nar1-CTC interaction site that cooperates with Nar1’s C-terminal tail to stabilize CCN complex formation.

### AlphaFold modeling of Nar1 and the CCN complex

Having defined two interfaces that work together to recruit the CTC to Nar1, we next used AlphaFold to generate structural models of Nar1 and the CCN complex (36). Although structures of several CTC subunits are available, both alone and in complex (Table S1) (16, 17, 18, 19, 20, 23), no experimental structures of Nar1 are available. Therefore, these AlphaFold models allow visualization of the Nar1-CTC interfaces and evaluation of whether the predicted Fe–S binding sites of Nar1 and the targeting complex are spatially compatible with our biochemical data and with Nar1’s proposed role as a Fe–S cluster carrier for the CIA pathway.

**Figure 5:**
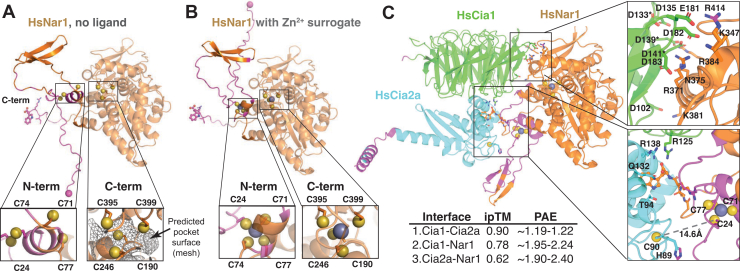
**AlphaFold modeling of Nar1 and the CCN complex.** All panels depict AlphaFold3 predicted structures of *Hs*Nar1 (*orange ribbon*) in the absence *(A)* and presence of two zinc ions *(B)* and the *Hs*CCN complex modeled with three zinc ions (*C*; *Hs*Cia1, *green ribbon*; *Hs*Cia2a, *cyan ribbon*). The *Hs*Nar1 N-terminus is a *magenta sphere* and its C-terminal divergent TCR peptide is shown as sticks. Regions of high uncertainty (pLDDT <50) are *magenta*. Putative Fe–S binding cysteines are *yellow* spheres. Modeled zinc ions are *gray spheres*. Insets in *Panel A* and *B* show detailed view of the AlphaFold3 predicted N-terminal (N-term) and C-terminal (C-term) cluster binding cysteine residues, with *gray* mesh representing the surface of the cavity predicted from the structural model to visualize the predicted pocket. Insets in *Panel C* highlight the AlphaFold3 predicted *Hs*Cia1 centered (*top*) and *Hs*Cia2-Cia2a (*bottom*) interfaces with *Hs*Nar1, with interface residues shown as sticks. Glutamates targeted for substitution are indicated with an *asterisk* (∗). The table lists the interfacial predicted template modeling and predicted aligned error scores for the binary interfaces, listed from highest confidence metrics to lowest confidence. TCR, targeting complex recognition.

We first examined the available AlphaFold models of *Ct, Sc*, and *Hs* Nar1 (37). All models revealed a well-folded ∼400-residue C-terminal domain and a lower-confidence N-terminal region (∼100 residues), consistent with the limited homology between Nar1 and [FeFe]-hydrogenase in this region (Figs. 5*A*, S7, and S8*A*). Examination of the eight conserved Cys residues proposed to ligate Nar1’s Fe–S clusters (Fig. 1, *B* and *C*) revealed that the C-terminal cysteines form a defined pocket compatible with metallocluster coordination (Fig. 5*A*) (25). In contrast, the N-terminal cysteines were distributed within a low-confidence region that did not define a clear cofactor binding site.

We reasoned that the absence of a bound cofactor might limit AlphaFold’s ability to organize this N-terminal region, as Fe–S clusters can serve as structural cores for small domains that lack extensive hydrophobic packing. Indeed, many Fe–S proteins, including cluster biogenesis factors, become unstable in their apo-state (38, 39, 40). Because AlphaFold3 does not support modeling Fe–S clusters as ligands, we explored whether inclusion of other metal ions could stabilize this region and help locate the putative cluster binding sites and increase the confidence scores in these regions. Although ferric iron can be specified as a ligand (41), its coordination chemistry differs substantially from that of Fe–S clusters. However, Zn^2+^ frequently occupies cysteine-rich coordination sites and Fe–S binding domains are sometimes misannotated as zinc fingers in which a single zinc ion is coordinated by the Fe–S cluster binding residues (42). These observations motivated us to include zinc ions as a Fe–S cluster surrogate and observed zinc coordination at both putative cluster binding sites with a position aligned error below 5 Å, and repositioning N-terminal cysteines into geometry compatible with metal coordination (Fig. 5*B*). However, neither inclusion of zinc nor Nar1’s binding partners (Cia1 and Cia2) resulted in folding of the N-terminal domain into a compact, globular domain (Figs. 5*C* and S8). These observations suggest that the N-terminal region of Nar1 may be conformationally dynamic, consistent with a role of a cluster carrier requiring transient metallocluster binding during cytosolic iron sulfur cluster assembly (25, 34).

Guided by our biochemical identification of a conserved acidic surface along blade 3 of the Cia1 β-propeller structure as a key Nar1 contacting region, we next examined the Nar1 models to identify a complementary interaction surface. We noticed that all AlphaFold models of Nar1 orthologs displayed a prominent, conserved basic region that is absent in [FeFe] hydrogenases (Fig. S9), consistent with a role in CTC recruitment. The electrostatic complementarity between this basic patch on Nar1 and the acidic surface on Cia1 provides a structural rationale for the strong salt-dependence of the Nar1-Cia1 interaction observed experimentally (Fig. 4).

We also noted that across all orthologs examined, AlphaFold3 predictions converged on similar CCN architecture (Figs. 5*C* and S8). These models predict that Nar1 engages the targeting complex through two interfaces (1): the C-terminal tail of Nar1 engaging the TCR peptide binding pocket at the Cia1-Cia2 interface and (2) the basic surface of Nar1 contacting the acidic region of Cia1 blade 3 (Figs. 5*C* and S10). The AlphaFold confidence metrics and predicted contact areas are consistent with our biochemical observations that Cia1-Nar1 is the primary interaction interface and Cia2 further stabilizes or facilitates the interaction. For example, in the *Hs*Cia1-Cia2a-Nar1 trimeric complex, the Cia1-Cia2a interface exhibits the highest confidence among the three pairwise interactions consistent with the availability of high-resolution structures of the fly Cia1-Cia2b complexes (20, 23). The Cia1-Nar1 interface, spanning approximately 1100-1500 Å^2^ across the AlphaFold3 predicted complexes, is also well-supported as judged by the interfacial predicted template modeling (ipTM = 0.78) and predicted aligned error (PAE values of 1.95–2.24 Å) confidence scores despite the absence of experimentally determined Cia1-Nar1 complexes from any organism (Fig. 5*C*). By comparison, the AlphaFold3 predicted *Hs*Cia2a-Nar1 interface is substantially smaller in both predicted buried surface area and confidence scores (ipTM ∼0.62; PAE ranging from ∼2.0–2.4 Å), consistent with the mid-micromolar dissociation constant for the *Ct*Cia1-Cia2-SPC-Nar1 complex.

Notably, independently generated AlphaFold model using the Nar1 paralog found in some animals, NarF, also converged on similar CCN complex architectures (Fig. S8, *D* and *E*) (10, 14). To our knowledge, there is no experimental evidence linking NarF to the CIA machinery, but it is notable that NarF maintains the eight proposed Fe–S binding cysteines, the basic patch proposed to anchor Nar1 to the CTC, and the divergent TCR motif (Figs. S7 and S8*D*). The retention of these features indicates NarF could recruit the CTC, consistent with the AlphaFold model of the *Hs*Cia1-Cia2b-NarF complex, but the biological implications of this observation remain unclear.

Animals also contain two Cia2 paralogs, called Cia2a and Cia2b (10). The *Hs*Cia2b paralog can also be readily modeled into a CCN complex, consistent with its proposed role as general CIA targeting factor (10). To evaluate whether this CCN complex can be accommodated within the full CTC, we superimposed the AlphaFold generated *Hs*Cia1-Cia2a-Nar1 model on the experimentally determined CTC crystal structure (PDB:6TC0) (20). This comparison suggested that Nar1 can be accommodated within the monomeric CTC assembly, containing one copy each of Cia1, Cia2, and Met18, but would sterically clash with the dimeric CTC arrangement observed in the crystal lattice (Fig. S8*F*). This observation is consistent with previous structural studies indicating that the monomeric CTC represents the physiologically relevant complex for client recognition (20). Our AlphaFold modeling also suggests the CTC monomer is the physiologically relevant complex for interaction with Nar1.

We noted that in all of the CCN models the flexible N-terminal region of Nar1 is positioned near the proposed Fe–S acceptor site, defined by His89 and Cys90 (*Hs*Cia2a numbering; Fig. 5*C*) (43, 44). Although these AlphaFold models and our biochemical studies herein do not directly address the mechanism of metallocluster transfer, the spatial proximity between the predicted Nar1 N-terminal cluster binding cysteine residues (the putative Fe–S cluster donor site) and the proposed cluster accepting residues of Cia2 suggests the bipartite Nar1 docking could be important for proper positioning of the proteins during cluster transfer.

To probe the importance of this cooperative bipartite docking, we altered the relative positioning of *Hs*Nar1’s two CTC interaction sites, shortening the region connecting TCR motif to its globular C-terminal domain. These truncations markedly reduced recovery of *Hs*Nar1 with *Hs*Cia1-Cia2a in affinity copurification assays (Fig. S11), indicating that the precise three-dimensional arrangement of these two CTC binding sites facilitates productive Nar1 binding to the targeting complex. Together, the models provide a structural framework that integrates our biochemical observations and supports a model in which Nar1 engages the CTC *via* two distinct binding interfaces that collaborate to stably bind Nar1 to the CTC. This bipartite interaction anchors Nar1 to the targeting complex while orienting its conformationally flexible N-terminal region toward the cluster binding surface of Cia2, providing a plausible structural arrangement for productive engagement of the CIA targeting complex during cytosolic Fe–S protein maturation.

## Discussion

In this study, we define the molecular basis by which Nar1 engages the CIA targeting complex (CTC) through a bipartite binding mode. Using cross-species comparisons together with qualitative and quantitative binding analyses, we demonstrate that Nar1 is recruited through two distinct interfaces: a primary, predominantly electrostatic interaction that anchors Nar1 to the conserved acidic surface lining Cia1’s third β-propeller domain (16, 20), and a secondary interaction in which Nar1’s C-terminal tail, bearing a divergent targeting complex recognition (TCR) motif, engages a peptide binding pocket at the Cia1-Cia2 interface (22). Together with AlphaFold-based modeling, these data provide a structural framework that reconciles prior conflicting models for Nar1 recruitment and rationalizes how formation of the Cia1-Cia2-Nar1 (CCN) complex could position the N-terminal Fe–S binding domain of Nar1 adjacent to the CTC cluster binding site.

A primary outcome of this work is resolution of longstanding discrepancies regarding the minimal requirements for Nar1’s recruitment to the targeting complex. Earlier studies suggested Nar1 interacts primarily with Cia1 (29), whereas later work concluded that the assembled Cia1-Cia2 complex is required (27). Our results reconcile these contradictory conclusions by showing that Cia1 provides the principal recruitment interface, while Cia2 enhances affinity through creation of the secondary Nar1 binding site situated at the Cia1-Cia2 interface. This cooperative mechanism ensures that stable Nar1 association only after formation of a functional CTC core, comprising Cia1 and Cia2, preventing premature interaction with isolated Cia1 which lacks Fe–S cluster binding residues. Notably, modeled CCN assemblies presented herein position the Nar1 N-terminal Fe–S cluster binding domain adjacent to the conserved, reactive Cia2 cysteine residue (Cys90 of *Hs*Cia2a) (15, 43). This quaternary structure is consistent with previous studies indicating that the N-terminal region of Nar1 is consistent with proposals that the N-terminal region participates in cluster trafficking (25, 27). We speculate this bipartite interaction serves to properly orient the putative Fe–S donor and acceptor sites within the CCN complex in a manner that is compatible with cluster transfer (20, 21).

The structural models further suggest that Nar1 represents an evolutionary repurposing of a hydrogenase-like scaffold for a metallochaperone function in the CIA system. Although Nar1 retains cysteine motifs characteristic of [FeFe]-hydrogenases, it lacks the catalytic H-cluster and instead has acquired new features, such as the basic patch implicated in CTC recruitment. The absence of this electrostatic surface in [FeFe]-hydrogenases suggests Nar1 has acquired this new protein-protein interaction module to facilitate its role as a trafficking factor with the CIA pathway.

Our findings further demonstrate that Nar1 engages a conserved, client-recruitment interface of the CTC. Previous structural studies implicated a negatively charged surface along the side of Cia1’s third β-propeller domain in facilitating client recognition (16, 20). Our biochemical analysis pinpoints this same region, the dominant, Cia2-independent interaction site for Nar1 recruitment. Disruption of this acidic surface selectively weakens CCN formation without impacting Cia2 binding or TCR peptide recognition, indicating that it functions as an organizational hub coordinating interactions with the Nar1 cluster donor and CIA client protein acceptors. The use of shared docking interfaces to coordinate interactions with multiple binding partners represents a recurring organizational principle in Fe–S cluster biogenesis pathways. For example, the bacterial cysteine desulfurase IscS engages its various sulfur acceptors IscU and TusA *via* overlapping binding sites (45, 46). Similarly, frataxin and ferredoxin compete for binding to IscU, tuning the rate of Fe–S cluster assembly by the ISC scaffolding complex (47). The parallels suggest that competitive engagement of a common recruitment interface might similarly function in CIA to coordinate exchange of an Fe–S cluster between the CTC and the Nar1 donor and a downstream CIA client acceptor. Together with previous studies identifying this same surface in client recognition, these findings suggest that this region of the CTC functions as a central recruitment hub that coordinates sequential interactions with cluster donors and client proteins.

Bioinformatic analyses of Nar1 and [FeFe]-hydrogenases pinpointed Nar1’s acquisition of a TCR peptide tail as a second functionally important divergence between Nar1 and hydrogenase proteins. We reveal that Nar1 engages the same TCR peptide binding client pocket exploited by CIA clients with canonical TCR motifs, but through a noncanonical interaction. Bioinformatic analysis demonstrated that the C-termini of Nar1 diverge from those of clients bearing a canonical TCR motif primarily at the penultimate position, where an acidic residue typical in CIA clients is frequently replaced by a basic residue and the antepenultimate bulky aliphatic residue is replaced with the small valine. Since the CTC binds Nar1 and clients through a shared interface, we speculate the divergence of their TCR motifs may allow the targeting complex to distinguish between Fe–S cluster donors and client acceptors. Consistent with this divergence, the recognition of Nar1’s C-terminus depends predominantly on the Cia2 arginine hotspot residue rather than contributions from both Cia1 and Cia2 as required for canonical TCR peptide binding (22). The proximity of the TCR peptide binding pocket to the proposed Fe–S binding site on Cia2 further suggests that TCR peptide engagement may influence cluster transfer.

A major barrier to investigations of cluster transfer between Nar1 and the CTC is the difficultly isolating Nar1 in a defined state, as previous cell-based studies have found the interactions investigated herein are regulated by iron (28). As isolated, the Nar1 preparations used herein contain 3 to 4 irons per polypeptide, in agreement with work by others (25, 27, 34). In an attempt to create fully apo-forms of Nar1, we attempted to mutate the Fe–S cluster binding cysteines but found that deletion of just one ligand did not prevent iron association and, consistent with the reports of others, additional substitutions disrupted Nar1 folding and thus precluded its use in biochemical investigations (25, 27). Similarly, attempts to load Nar1 *via* chemical reconstitution resulted in incomplete cluster incorporation. We note that it was recently reported that *Sc*Nar1 could be reconstituted with two [4Fe–4S] clusters and one [2Fe–2S] cluster, but the ability of Nar1 to donate one or more of these clusters to the CTC was not investigated in that or any other system.

The bipartite recruitment mechanism defined herein provides a structural framework for understanding how Nar1 engages the CIA targeting complex and lays the foundation for future mechanistic studies investigating how the CTC regulates interaction with the Nar1 donor and the CIA client acceptors during cytosolic Fe-S protein maturation. A key remaining challenge is to obtain clear *in vitro* evidence for Nar1’s proposed Fe–S donation activity and, more broadly, how iron modulates the complex network of interactions in the CIA pathway (28). Given the emerging links between defects in cytosolic Fe–S biogenesis and human disease (6, 7, 11, 12), defining how cluster exchange occurs within the CTC-Nar1 complex represents an important next step to understanding how the disruption of this process contributes to cellular dysfunction.

## Experimental procedures

### Cloning

The plasmids for the heterologous expression of *C. thermophilum* (*Ct*) and *H. sapiens* (*Hs*) Nar1, with N-terminal His-SUMO tag, were created by insertion of the codon optimized gene into pTB146, analogous to heterologous overexpression plasmids for *Sc*Nar1 (35). The plasmid for the heterologous expression of *Hs* double-tagged Cia1, with N-terminal His-TEV-Strep tag, were created by insertion of the codon optimized gene into pDUET1. Variants, including *Ct*Nar1^W609A^, *Hs*Nar1^Δ10^ (deletion of residues 462–471), and *Hs*Nar1^Δ15^ (deletion of 457–471) and *Hs*Nar1^ΔTCR^ (deletion of residues 472–476) were generated using Q5 Site-Directed Mutagenesis Kit (New England Biolabs). The SPC Nar1 was made by inserting nucleotides encoding the last 10 amino acids of *Ct*Nar1 at the C-terminus of SUMO (21, 22).

The vectors for expression of *Ct*Cia1-Cia2 (co-expressed from pDUET1 plasmid), *Hs*Cia1, and *Hs*Cia2a (with deletion of the first 27 residues, ΔN27, and a GST-tag) were previously reported (18, 21, 22). The *Ct*Cia1 charge-swapped variant (3E>K: E187K, E191K, E193K) and *Hs*Cia1 charge-swapped variant (D/E>K: D13K, E139K, E141K) were created by Q5 mutagenesis. Successful creation of all constructs was verified by DNA sequencing.

### Expression and purification

Expression and purification of *Ct*Cia1-Cia2, *Hs*Cia1-Cia2a and SPC-Nar1 were carried out as described (21, 22). Nar1 (*Ct* or *Hs* WT proteins or their variants) was expressed into BL21(DE3) grown at 37 °C to an OD_600_ of 0.7 − 0.8. The temperature was then lowered to 30 °C and IPTG (0.5 mM), ferric ammonium citrate (25 μM), and L-cysteine (2 mM) were added. The cells were collected 3.5 to 4 h after IPTG addition.

For aerobic purification of *Ct*Nar1, cell paste (∼7 g) was resuspended with 70 ml Buffer A [50 mM NaPO_4_ (pH 8.0), 100 mM NaCl, 5% glycerol, and 5 mM BME at 4 °C] supplemented with 0.5% octylthioglucoside (GoldBio), 5 mM imidazole, 1 mM PMSF (GoldBio), protease inhibitor tablet (Thermo Fisher Scientific), 4 kU DNase nuclease (Thermo Fisher Scientific). rLysozyme (MilliPore Sigma) (45kU/g) was added and cells were disrupted by sonication. The clarified lysate was added to 1 ml of Ni-NTA resin which was washed with Buffer A supplemented with imidazole as follows, 40 column volumes (CV) with 5 mM imidazole, 20 CV with 10 mM imidazole, and 20 CV with 20 mM imidazole before elution of the column with Buffer A containing 300 mM imidazole. Protein containing fractions were combined and concentrated using Amicon ultra centrifugal filter units (10 kD MWCO, Millipore Sigma). Imidazole was removed by gel filtration using a PD10 column (Fisher Scientific) equilibrated in Buffer A. The protein was concentrated to 20 to 30 mg/ml and stored at −80 °C.

For anaerobic purification of *Hs*Nar1, the cell pellet (∼6–8 g) was introduced into the glovebox (Coy Labs). Purification was carried out as described for the aerobic purification of *Ct*Nar1, except cells were not sonicated, Buffer B [50 mM Tris (pH 8.5), 250 mM NaCl, 10% glycerol, and 5 mM BME] replaced Buffer A, and 2 ml Ni-NTA resin was used. *Hs*Nar1 was concentrated with an Amicon ultra centrifugal filter unit (30 kD MWCO) before storage at −80 °C.

For aerobic purification of *Ct*Cia1-Cia2-Nar1 complex for SEC analysis, *Ct*Cia1-Cia2 and *Ct*Nar1 were separately expressed in BL21 (DE3) cells as described above before combining cell pastes for *Ct*Cia1-Cia2 (4 g) and *Ct*Nar1 (6 g). The mixed cells pastes were resuspended in Buffer C [50 mM CHES (pH 9.0), 100 mM NaCl, 5% glycerol, 1 mM DTT; final volume 100 ml], supplemented with 1 mM PMSF, protease inhibitor tablet, 4 kU DNase nuclease and 5.0 g of CellLytic Express (Sigma-Aldrich). After 45 min incubation at 4 °C with gentle stirring, the extract was clarified by centrifugation, and the soluble fraction was added to 2 ml Strep-Tactin XT Superflow resin (IBA Lifesciences). The column was washed with 15 to 20 CV of Buffer C and the protein was eluted with Buffer C supplemented with 30 mM D-Biotin (IBA) and 40 mM NaOH. Protein containing fractions were combined and concentrated using Amicon ultra centrifugal filter units (10 kD MWCO), and the buffer was exchanged into 50 mM CHES pH 9, 100 mM NaCl, 2% glycerol, and 1 mM DTT by overnight dialysis. The protein concentration was determined using Assay Dye Reagent Concentrate (Bio-Rad) assuming a molecular weight of 154.5 kDa for the 1:1:1 complex, including tags. The protocol for isolation of *Ct*Cia1-Cia2 for size exclusion chromatography (SEC) analysis was the same as the one described above for the *Ct*Cia1-CIa2-Nar1 complex but using the cell expressing *Ct*Cia1-Cia2 only.

For purification of *Hs*Cia1-Cia2a-Nar1, TEV protease treated *Hs*Cia1-Cia2a (TEV removes His-GST tag from *Hs*Cia2 and the His-tag from *His*Cia1, leaving a Streptactin tag on *Hs*Cia1 for affinity purification) was mixed with *Hs*Nar1 in a ratio of 1 μg *Hs*Cia1-Cia2a for every 2 μg *Hs*Nar1 in Buffer D [50 mM Hepes (pH 8.1), 100 mM NaCl, 5% glycerol]. After 1 h at 4 °C, the CCN complex was purified *via* streptactin chromatography.

### Iron and sulfide content determination

Iron content was determined by ferrozine assay (48). Briefly, three 30 μl aliquots of Nar1 (∼25–30 μM) or buffer-only were supplemented with 70 μl of 2M HCl, boiled, and centrifuged to remove precipitated protein. The supernatant was reserved, pellet was washed with 100 μl of 2M HCl, boiled, centrifuged and supernatant was combined with the reserved sample. Water (360 μl), 75 mM sodium ascorbate (40 μl) and 10 mM ferrozine (200 μl) were added and vortexed. Saturated ammonium acetate (200 μl) was added and the solution was vortexed again. The absorbance at 562 nm was then used to determine the iron concentration, using ferrozine’s extinction coefficient of 27,900 M^−1^ cm^−1^.

Analysis of acid-labile sulfide was determined by the method of Rabinowitz (49). Nar1 (∼10 μM in 200 μl) or buffer control were analyzed in triplicate. Fresh zinc acetate (600 μl of a 1% solution) was added, followed by sodium hydroxide (30 μl of 12% solution). After incubation with gentle stirring for 45 min, *N,N*-dimethyl-p-phenylenediamine (150 μl of 0.1% solution) added to the bottom of the tube then ferric chloride (30 μl of 23 mM) was added to the to the top layer. The samples were stirred vigorously for 15 min, centrifuged for 20 min and supernatant was analyzed for absorbance 670 nm, using the extinction coefficient of the methylene blue product 34,500 M^−1^ cm^−1^.

As isolated, the *Ct*Nar1 contained 2.8 ± 0.35 iron atoms and 2.6 ± 0.54 acid labile sulfur per polypeptide (n = 3). *Hs*Nar1 contained 3.7 ± 1.23 iron atoms and 4.0 ± 0.54 acid labile sulfur per polypeptide (n = 3).

### Size exclusion chromatography analysis

All steps were carried out at 4 °C with a Superdex 200 5/150 Gl column (GE Healthcare) and eluted with the indicated buffer at a flow rate of 0.4 ml/min. *Ct*Cia1-Cia2-Nar1 or *Ct*Cia1-Cia2 samples were diluted to 3.5 mg/ml in SEC-CHES buffer [50 mM CHES (pH 9.0), 100 mM NaCl, 2% glycerol, and 1 mM DTT]. The samples (200–300 μl) were centrifuged immediately before analysis.

Samples of *Hs*Nar1-Cia1-Cia2a, *Hs*Cia1-Cia2a, and the individual subunits were diluted to 1 to 1.5 mg/ml with SEC-Hepes buffer [50 mM Hepes (pH 8.1), 100 mM NaCl, 5 mM BME, 5% glycerol] in a final volume of 200 μl and centrifugated to remove any aggregates.

The molecular weight of eluted complexes was estimated by plotting the gel phase distribution coefficient (K_av_) against the logarithm of the molecular weight standards (Gel Filtration Standard, BioRad). For SDS-PAGE analysis, fractions (1 ml) were collected, concentrated to 200 to 240 μl using Amicon ultra centrifugal filter units (10 MWCO) before gel electrophoresis.

### Affinity copurification analysis

Affinity copurification assays were carried out as previously described (21, 22). Briefly, strep-tagged *Ct*Cia1-Cia2 complex (strep tag on *Ct*Cia2) or strep-tagged *Ct*Cia*1* was mixed with an equimolar amount of *Ct*Nar1 in Buffer A (∼600–650 μl). After 1 h incubation, the mixture was batch absorbed to Strep-Tactin XT Superflow resin (200 μl) resin. The resin was collected, washed, and eluted with Buffer A supplemented with 50 mM D-Biotin. The resulting elution fractions and the corresponding input samples were analyzed *via* SDS-PAGE. For the human orthologs, the experiment was performed similarly except *Hs*Cia1-Cia2a (Strep-tagged Cia1, 100–200 μg) and *Hs*Nar1 (200–300 μg) were mixed in a one-to-two mass ratio in 50 mM Hepes (pH 8.1), 100 mM NaCl, 5% glycerol.

### Fluorescence anisotropy binding assay

Equilibrium binding assays with *Ct*Cia1-Cia2 complex and FITC-HLDW probe were carried out as described (22). Briefly, *Ct*Cia1-Cia2 (3 μM) or *Hs*Cia1-Cia2a (10 μM) was mixed with FITC-HLDW (0.05 μM) in the Buffer A supplemented with 0.1 mg/ml BSA and unlabeled competitor (Nar1, 0–30 μM; SPC-Nar1, 0–150 μM) in a final volume of 200 μl. The anisotropy values were recorded using a SpectraMax5 plate reader (MolecularDevices) In some experiments, the NaCl concentration was raised to 700 mM to determine how the ionic strength affects binding affinity.

### Nano differential scanning fluorimetry

*Hs*Cia1, either the WT or the D/E > K variant, were diluted to 0.3 to 0.5 mg/ml in 50 mM Hepes (pH 8.1), 100 mM NaCl, 5% glycerol. The samples (∼10 μl) were loaded into standard grade capillaries (NanoTemper) and analyzed using a Prometheus nano differential scanning fluorimetry (NT.48 Series, NanoTemper). The temperature was increased from 20 °C to 95 °C at a ramp rate of 1 °C per minute while monitoring the fluorescence intensity at 330 nm and 350 nm. The melting temperature (*T*_*m*_) was calculated from the first derivative of the fluorescence ratio 350 nm/330 nm using the PR.ThermControl software (NanoTemper). All measurements were conducted in triplicate and are reported as the average ± the standard deviation.

### Bioinformatic analysis of Nar1’s TCR peptide

Eukaryotic sequences harboring the cytosolic Fe–S cluster assembly factor (IPR050340) were downloaded from InterPro (https://www.ebi.ac.uk/interpro/). Only sequences between 200-1000 residues were retained before clustering of sequences into groups with ≥70% sequence homology and retaining one representative sequence of each group. The remaining sequences were aligned and manually processed to remove those missing any of the eight conserved cysteine residues or those not terminating in an aromatic residue. The last three residues of the remaining 631 Nar1 sequences were analyzed as described (21), with enrichment factors calculated as frequency of each amino acid at a given position normalized by its frequency in the human proteome.

### AlphaFold modeling

For Nar1 structures in the absence of zinc, AlphaFold models were downloaded from the AlphaFold2 database. For modeling of *Hs*Nar1 bound to zinc, the AlphaFold3 server was used, including four zinc ions as ligands. For figures, only zinc ions with PAE values below 5 Å, corresponding to zinc ions bound at the N-terminal and C-terminal Fe–S sites, were displayed. The highest confidence structure of *Hs*Nar1 along with the AlphaFold confidence and MSA outputs are included as supporting data.

For structures of the CCN complex, three zinc ions were used along with one copy of each of Cia1, Cia2 and Nar1. All AlphaFold output files of CCN complexes, including the 5 most likely models, paired and unpaired alignments and confidence scores are included as supporting data files. The ConSurf PyMOL sessions for the *Hs*Cia1-Cia2a-Nar1 highest confidence structure were created using multiple sequence alignments provided in supporting information and visualized using the PyMOL Molecular Graphics version 3.1.6.1 (Schödinger, LLC) (50). The top-ranked (model_0) AlphaFold generated models of *Hs*Nar1 in the presence and absence of four zinc ligands, and the *Hs*Cia1-Cia2a-Nar1 complex in the presence of three zinc ions along with paired and unpaired MSA files and confidence metrics including ipTM and PAE scores are included as supporting data.

## Data availability

All data generated or analyzed during this study are included int his published article and its Supporting Information files, except for AlphaFold-generated models. The Nar1 AlphaFold2 generated models were downloaded from the AlphaFold database using fourth version of the database-curated models. For the zinc-bound models of Nar1 and the Cia1-Cia2-Nar1 complexes shown in the main text, the top scoring human HsNar1 model containing four zinc ligands and the top-scoring human HsCia1-Cia2a-Nar1 complex model containing three zinc ions, together with associated confidence metrics and summary data, are provided as Supporting Data.

## Supporting information

This article contains supporting information.

## Conflict of interest

The authors declare that they have no conflicts of interest with the contents of this article.
